# Local Neuronal Responses to Intracortical Microstimulation in Rats' Barrel Cortex Are Dependent on Behavioral Context

**DOI:** 10.3389/fnbeh.2022.805178

**Published:** 2022-03-22

**Authors:** Sergejus Butovas, Cornelius Schwarz

**Affiliations:** Systems Neurophysiology, Werner Reichardt Center for Integrative Neuroscience, Hertie-Institute for Clinical Brain Research, University Tübingen, Tübingen, Germany

**Keywords:** intracortical electrical stimulation, behavioral modification, head-restraint rat, barrel cortex, sensory cortical prosthesis

## Abstract

The goal of cortical neuroprosthetics is to imprint sensory information as precisely as possible directly into cortical networks. Sensory processing, however, is dependent on the behavioral context. Therefore, a specific behavioral context may alter stimulation effects and, thus, perception. In this study, we reported how passive vs. active touch, i.e., the presence or absence of whisker movements, affects local field potential (LFP) responses to microstimulation in the barrel cortex in head-fixed behaving rats trained to move their whiskers voluntarily. The LFP responses to single-current pulses consisted of a short negative deflection corresponding to a volley of spike activity followed by a positive deflection lasting ~100 ms, corresponding to long-lasting suppression of spikes. Active touch had a characteristic effect on this response pattern. While the first phase including the negative peak remained stable, the later parts consisting of the positive peak were considerably suppressed. The stable phase varied systematically with the distance of the electrode from the stimulation site, pointing to saturation of neuronal responses to electrical stimulation in an intensity-dependent way. Our results suggest that modulatory effects known from normal sensory processing affect the response to cortical microstimulation as well. The network response to microstimulation is highly amenable to the behavioral state and must be considered for future approaches to imprint sensory signals into cortical circuits with neuroprostheses.

## Introduction

The whisker-related tactile sense in rodents is an exquisite example of active sensing and perception. Rats typically acquire tactile information about their environment by actively sweeping their array of whiskers across the object of interest (i.e., active touch). Sometimes, however, they make contact with a moving object when the whiskers are at rest (i.e., passive touch). These two modes of tactile processing are characterized by different modulation of signal representation in the barrel cortex (whisker representation of the primary somatosensory cortex in rodents) (Fanselow and Nicolelis, 1999; Crochet and Petersen, 2006; Ferezou et al., 2006; Hentschke et al., 2006; Lee et al., 2008; Chakrabarti and Schwarz, 2018). The phenomenon has been observed in several animal species and humans and is often called “sensory gating” (Chapman et al., 1987, 1988; Gertz et al., 2017). The cardinal response of barrel cortex neurons to a transient whisker deflection is an action potential or two followed by a strong inhibitory period (Simons, 1978; Stüttgen and Schwarz, 2008). The abovementioned studies of sensory gating in the whisker system have uniquely shown that both phases of the sensory response in the barrel cortex are suppressed when the whiskers are actively moved (“gating”). Sensory gating is independent of the activity that may be evoked in the whisker follicle during whisking (Leiser and Moxon, 2007; Khatri et al., 2009) and persists after interruption of the infraorbital nerve (Hentschke et al., 2006; Poulet and Petersen, 2008). It is further presented on the tactile pathway as early as the trigeminal nuclei, holding its first synaptic station. Sensory gating has been shown to be selective for ascending channels, being prominent in the lemniscal pathway, while absent in the extralemniscal pathway (Chakrabarti and Schwarz, 2018 for review of pathways see Feldmeyer et al., 2013). The same study has shown that corticofugal projections play a critical role for sensory gating on subcortical stations of the tactile pathway. A lesion of primary somatosensory cortex and surrounding parietal cortical areas abolishes sensory gating.

In future cortical neuroprostheses, microstimulation is intended to substitute for ascending sensory signals. The question, therefore, arises whether and how the activity imprinted directly into the neocortex is affected by context-dependent changes. In this study, we used sensory gating as a model case to manipulate the behavioral context and concomitant neuronal activity of the cortical origin to study how the short latency, local cortical effects of intracortical microstimulation are modified. To this end, we used head-fixed rats that were operantly conditioned to move a whisker in a goal-oriented way. We found that the short-latency parts of the local response, including the first excitatory volley of spikes, corresponding to a short-latency negative deflection of the local field potential (LFP), is stable across context, while the parts of the response with longer latency, characterized by strong firing rate suppression, is amenable to modulation by active touch.

## Materials and Methods

### Surgical Procedures and Behavioral Training

Three male Long Evans rats (12–14 weeks old, bodyweight 350–450 g) were used in this study. All experimental and surgical procedures were performed in accordance with the guidelines of animal use of the Society for Neuroscience and German Law (approval of Regierungspräsidium Tübingen). All the animals were accustomed to the experimenter and behavioral setup for at least 2 weeks before surgery. Surgery was performed to implant electrode arrays and the post for head fixation. Anesthesia was initialized with ketamine/xylazine (100 mg/kg/15 mg/kg i.p.) and was continued with isoflurane. Isoflurane concentration (1–2.5%) was adjusted to keep the painful hind paw reflex below the threshold. The animal's body temperature was held at 37°C by a feedback-controlled heating pad (Fine Science Tools, Heidelberg, Germany). The rat was mounted on a stereotaxic apparatus, and craniotomy over the barrel cortex was performed (coordinates P2–3/L4.5–5.5). A set of stainless-steel microscrews (Morris Co., Southbridge, MA, USA, part number 0x1/8 flat) were placed in the skull. The electrode array with vertically movable electrodes (refer to the Section Results) was implanted into the barrel column of whisker C1 (determined by mapping out the surface of the cortex by deflecting the whisker with a handheld cotton swab and recording spike and LFP responses *via* a single microelectrode) and embedded together with the skull screws into light-curing dental cement (Flowline, Heraeus Kulzer, Hanau, Germany). The wound was cleaned and disinfected with hydrogen peroxide at the end of the surgery. The open skin was sutured and carefully attached to the implant. In one animal, the infraorbital nerves on both sides of the head were cut as done before (Hentschke et al., 2006). To this end, the skin was shaved in the region of the face below the eyes. The skin was incised, and the nerve which is hidden below the musculature was prepared. The nerve was isolated from the underlying tissue and cut in its entirety after two sutures had been noosed around it. The nerve stumps were sutured in place leaving a gap of minimally 1 mm to prevent regeneration. The skin in the face was disinfected and sutured carefully. After the surgery, animals were kept warm and treated with analgesics (2 injections caprophen, 5 mg/kg, s.c.), they were allowed to recover for 14 days.

Rat housing, handling, accommodation to head fixation, and water control were performed as described before (Schwarz et al., 2010). Training sessions were scheduled two times a day for 5 days a week followed by 2 days of free access to water. All behavioral experiments were conducted inside a dark experimental box clad with sound-absorbing foam. The animals were monitored using infrared cameras. The behavioral training consisted of two phases. At first, the subjects were conditioned to respond to intracortical electrical stimulation by emitting a lick within an interval of 0.5 s after stimulus presentation to obtain a drop of water as a reward. Initially, the animals received a train of stimuli consisting of 15 pulses (at 320 Hz) at a suprathreshold intensity of 3.2–4.8 nC (Butovas and Schwarz, 2007). Interstimulus intervals were randomly varied from 3.25 to 6.25 s (mean 5 s, flat probability distribution). As soon as the animals responded well to the high-intensity stimuli, the number of pulses was reduced, guided by their performance until the animals were able to respond reliably to single pulses. To discourage premature responses, a 1 s interval before stimulus presentation was introduced, in which, a lick would delay the stimulus presentation by a new randomly drawn interstimulus interval.

**Figure 1:**
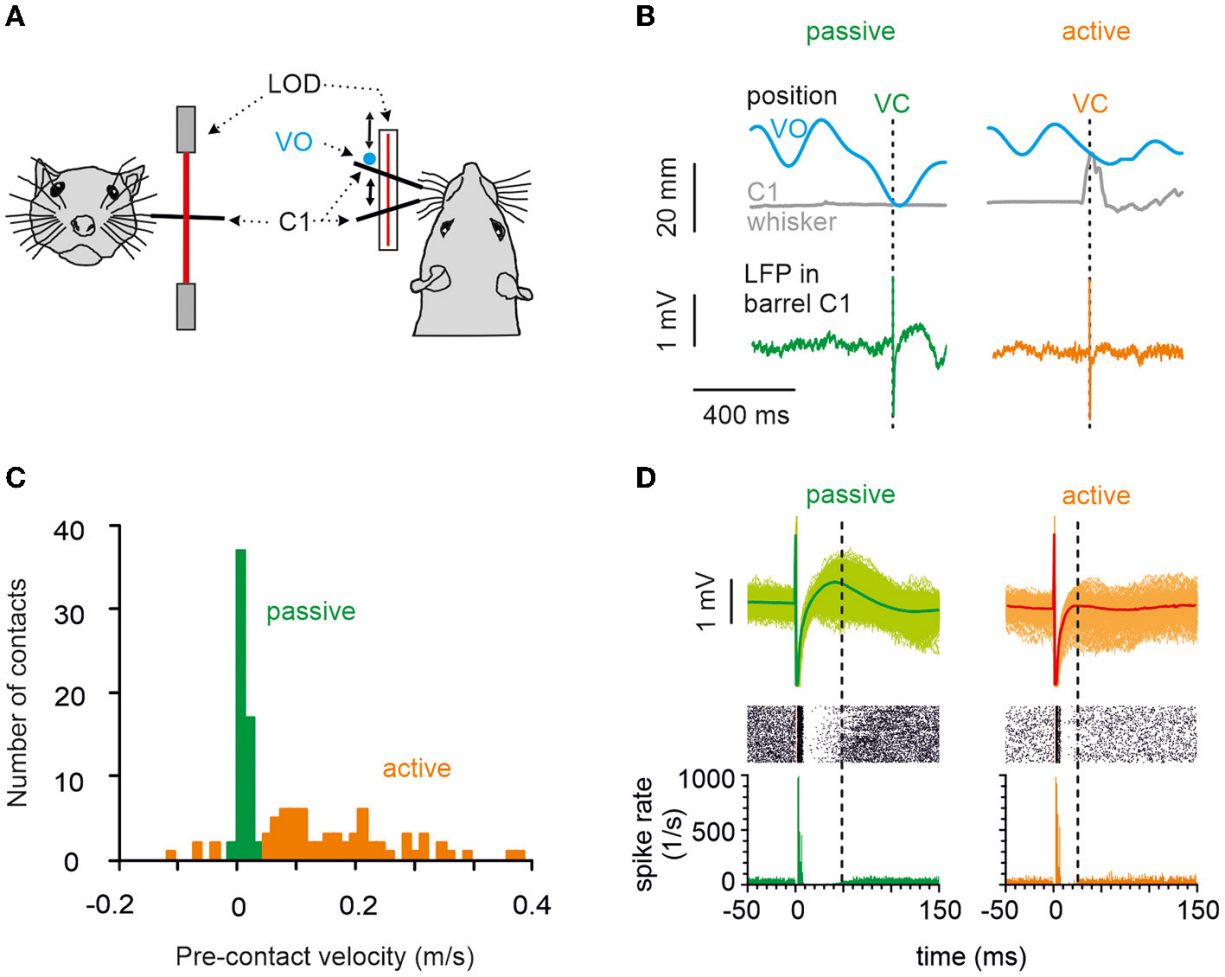
Behavior, the principal measurements, and classification of touches into active vs. passive. **(A)** A head-fixed rat's whisker C1 was equipped with a light polyimide tube and tracked in real-time using a laser optical device. The movement of a virtual object (VO) was modeled in a computer and compared in real-time to the whisker position. **(B)** When measured whisker trajectory and modeled VO movement trajectory crossed, a VC occurred (realized as microstimulation in barrel column C1). Two VCs (arrows), one passive (left) and one active (right), are shown. Gray: the trajectory of whisker C1. Blue: the trajectory of the VO. Bottom: examples of LFP responses in barrel cortex with passive (green) and active (orange) VO contacts. **(C)** Histogram of whisker mean velocity distribution, measured within the 10 ms interval preceding the VCs obtained from an example session. Precontact velocities, classified as passive and active, are marked in green and orange, respectively. **(D)** Circumstantial observation of spikes (obtained in one animal in one session) allows a glimpse of how features in LFP signals relate to those in cortical spikes rates. Bottom: Spike density of spike responses to passive (green) and active (orange) contacts. Note that the inhibitory period was longer and more pronounced after passive contacts, while excitation remained unaffected (marked by the vertical broken line on the right of each plot). Center: Raster display. Top: Overlay of cutouts of the LFP responses to passive (green traces) and active (orange traces) contacts recorded from the same electrode as the spikes shown below. The thick trace is the average evoked LFP. The slow positive deflection amplitude reflects the strength and duration of the inhibitory response as seen in the spike density. Time of virtual contact is at time 0.

Before the second phase of behavioral training, whiskers on both sides were cut to a length of 2 cm. Before each session, a polyimide tubing (250 μm in diameter, 3 cm length, and 0.7 mg weight) was slipped onto whisker C1. The movement of the whisker at ~2.5 cm distance from the face was monitored using laser illumination from above, and the detection of the whisker's shadow on a linear CCD (Figure 1A) located below the whisker (Bermejo et al., 1998) was carried out. The rostro-caudal component of whisker movements was tracked at a temporal resolution of 2.5 kHz and a spatial resolution of 0.4 μm (Metralight Inc., San Mateo, CA, USA). The rats were trained on a motor task that consisted in moving the C1 vibrissa in the rostro-caudal direction to find a virtual object (VO). The VO was computer-simulated at a resolution of 1 kHz and could be made to move on any arbitrary trajectory in the rostro-caudal direction. Online comparison of the real trajectory of the whisker and virtual trajectory of the VO yielded virtual contacts (VC, i.e., crossing of the two trajectories), leading to a water reward. The whisker movement was exclusively in the air without any “real” contact with objects. Thus, from the view point of the animal, a VC and water reward happened at varying times and positions, and its probability would be greatly increased by active whisker movements. To shape the rats' behavior, we started with a stationary VO—first positioned just rostral to the resting point of the whisker. With an increasing success rate of the rat to generate VCs, the stationary VO was gradually moved in the rostral direction. At this stage, the rat had to move the whisker forward from the resting point to generate a VC. Once VCs were regularly generated, the VO was set in motion and moved on a trajectory of low-pass-filtered Gaussian noise (limiting frequency 10 Hz) and maximal amplitude of ~3 cm (just reaching the resting point of the whisker). At this stage, therefore, “passive” VCs, with a stationary whisker positioned at the resting point was possible. VCs of the whisker with the VO triggered an immediate barrel cortex microstimulation, given the last lick and the last VC both occurred more than 1 s ago.

When rats regularly achieved VCs, they were moved to the data acquisition stage, in which all data presented here were sampled. During the data acquisition stage, we exclusively used a moving VO and single-pulse bipolar stimulation (i.e., negative first) at an intensity of 4.8 nC. The whisker trace, the VO, and the behavioral data (i.e., timestamps of stimuli, licks, VCs, and rewards) were computed, controlled, and recorded by a custom-made software running on a LabView Real Time System (National Instruments, Texas, USA).

### Microstimulation and Electrophysiology

Mobile microelectrode arrays were custom-made (Haiss et al., 2010). Shortly, nine pulled glass-coated platinum tungsten electrodes (i.e., 80 μm shank diameter, 25 μm diameter of the metal core, free tip length of 10 μm, and impedance > 1 MΩ; Thomas Recording, Giessen, Germany) were placed inside a 3 × 3 array of polyimide tubing with a distance of 300 μm (HV Technologies, Trenton, GA, USA). The electrodes were soldered to Teflon-insulated silver wires (Science Products, Hofheim, Germany), which, in turn, were connected to a microplug (Bürklin, Munich, Germany). The electrodes were attached to a rider that moved along the thread of a screw and, thus, allowed moving them into the cortex. The electrode array was inserted over barrel C1 of posteromedial barrel subfield, initially at the depth of 250 μm below the pia mater. All recordings presented in this study were carried out at a depth of 1,200 μm, roughly corresponding to layer 5. In all three rats, we assured that tactile responses in LFP (and if available spike responses) to rapid whisker deflections of whisker C1 were present throughout the array. Histological verification of stimulation sites was not performed. Electrical stimulation pulses were generated using a programmable stimulator (model number: STG 2008; MultiChannelSystems, Reutlingen, Germany). For microstimulation, a low-impedance electrode on one of the corners of the quadratic array was selected and used throughout training and experimental sessions. Simultaneous LFP signal recordings were performed using a multichannel extracellular amplifier (MultiChannelSystems, Reutlingen, Germany; gain 5,000, sampling rate 20 kHz).

### Whisker “Virtual Contact” Classification and Data Analysis

All analyses were performed using custom-written MATLAB (Mathworks Inc., Natick, MA, USA) scripts. A VC was defined as the crossing point of whisker and VO trace as performed earlier (Gerdjikov et al., 2013). Each VC was classified into the “active” (A) or “passive” (P) class depending on the precontact whisker trace. A detailed description of the analysis is presented elsewhere. In brief, we first differentiated the whisker movement trajectory to calculate a velocity trace. The first criterion was the instantaneous velocity. It had to exceed a threshold derived from the bimodal distribution of whisker velocities 5 ms preceding contact (refer to Figure 1C, Hentschke et al., 2006). The peak of whisker velocities around zero mirrors the spurious whisker movements at rest for each individual (denoted in green in Figure 1C). We fitted a Gaussian to the peak around zero and defined the velocities defining the double of its half-peak width as criteria, which when surpassed in positive or negative direction would indicate an active VC. Before classifying a VC as passive, a second criterion had to be matched: root mean square of whisker velocity during 75 ms before the contact had to be below 0.03 m/s (Hentschke et al., 2006).

**Figure 2:**
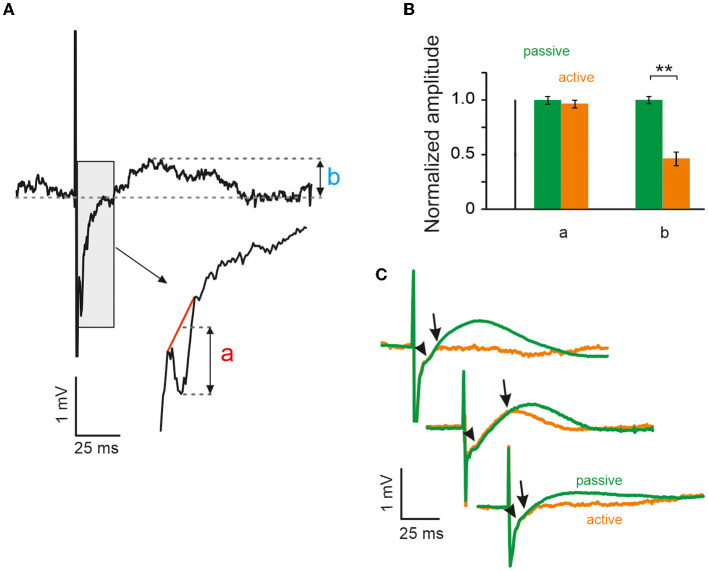
Whisking reduces evoked local field potential (LFP) activity during the inhibitory period but not during the excitatory period. **(A)** Example of LFP response, which demonstrates sharp negative deflection (a) followed by long positive wave (b), presumably corresponding to electrically evoked excitation and inhibition respectively (cf. Figure 1D). **(B)** Peak amplitudes of excitatory LFP response (a) to both active and passive contacts yielded no difference, unlike amplitude of the inhibitory period (b), which had higher maximum amplitude during passive contacts in all three animals (**significant using Mann-Whitney *U*-test, error bars CI95, see text). **(C)** Mean LFP responses to both passive (green) and active (orange) contacts in all three animals (the infraorbital nerve was cut in the animal shown on top).

The excitatory cortical response was imposed as a negative peak with a very stable latency of 2 ms after the stimulation (Figure 2A). To measure its amplitude, the negative peak was cut out from the LFP trace and substituted by linear interpolation. The difference between the maximum negative-peak amplitude and the corresponding value of the interpolation line was then taken as the measure of the peak amplitude (as shown in the inset in Figure 2A). Since the excitatory peak amplitudes varied considerably from animal to animal, the measurements were normalized to mean amplitude as observed after passive VC in each animal. The amplitude of the positive evoked LFP wave was simply its maximum peak value (Figure 2A).

Statistical inference was performed using a bootstrap procedure (1,000 resamples) that output 95% confidence intervals (CI95) of the distributions to be compared. For the comparison of LFP distributions along poststimulus time, we calculated the effect size of the two distributions as given by the area under the ROC curve (AUC). The AUC measures the probability of an ideal observer confronted with a random pick from the two response distributions to correctly classify it. AUC is 0.5 if the two distributions are identical and 1, if the two distributions are perfectly discriminable (Green and Swets, 1966). CI95 of the AUC readings were calculated by bootstrapping LFP amplitude distributions (1,000 resamples). All data are presented as mean [lower CI95, upper CI95], if not indicated otherwise.

## Results

We trained head-fixed rats to move their whiskers to find a nonstationary, computer-simulated VO. The rostro-caudal whisker movement was tracked and compared with the location of the VO. A crossing of the two trajectories was called a VC, which was immediately followed by the delivery of a drop of water and a microstimulation pulse in the rat's barrel cortex, given the rat did not lick at the spout in a time interval 1 s before the VC (Figure 1A). The result was that the rat whisked voluntarily in free air at its own pace and got stimulated—unpredictably for the rat—at different whisker positions and velocities that covered the entire kinematic range including rest. A VC was identified as “active,” if whisker movement preceded the contact and “passive” if the whisker was at rest. Figure 1B shows single trials, and Figure 1C shows an example session of passive and active contacts (refer to the Section Materials and methods for details). The quantitative analysis in this report is based on LFP recordings. Occasionally, however, we were able to record multiunit spike data along with the LFPs from the same electrodes. To show the correspondence of LFP recordings to spike recordings, Figure 1D plots an example session (*n* = 687 passive, *n* = 176 active trials) with LFP traces, raster plot, and peristimulus-time histograms (PSTHs). Spike responses in our awake rats showed the stereotypical cortical spike response pattern to electrical stimulus composed of a short excitatory peak followed by a long-lasting inhibition of tens of millisecond duration (as shown before under ketamine anesthesia: Butovas and Schwarz, 2003; Butovas et al., 2006). The corresponding LFP pattern was a short-latency negative LFP wave (not well seen in the average LFP of Figure 1D, refer to next paragraph and Figure 2) and a long-lasting LFP wave of positive polarity. Comparison of active vs. passive VCs (left vs. right side in Figure 1D) clearly shows a shortening of the spike suppression in the active case, which was reflected in the near-abolishment of the positive LFP wave (the right vertical broken line in each plot points to the end of the suppression period where spiking resumes again).

To assess the negative LFP deflection in quantitative detail, Figure 2A shows a single trace at higher temporal resolution. Typically, the negative peak was located on the part of the voltage trace that was on its upward rise after the strong negativity imposed by the stimulus artifact (gray box). The gray box is shown again at a shorter time scale and demonstrates how the amplitude (a) of the negative peak was quantified. Negative peak amplitudes (a) during active and passive VCs were very similar and, in fact, did not differ significantly. In contrast, the amplitude of the slow positive deflection (b) was markedly and significantly different between the two conditions. Population data (*n* = 3 rats) from the three recording electrodes neighboring the stimulation electrode are shown in Figure 2B. All data shown in this panel were normalized and rendered unitless by dividing each measured amplitude by the average amplitude observed in the passive condition (carried out separately for measures a and b). For the short-delay negative peak, (a) we analyzed *n* = 820 passive VCs (amplitude of 1 [0.038, 0.03]), and *n* = 289 active VCs (amplitude of 0.965 [0.037 0.036]). Testing the two means did not reveal a significant difference (Mann-Whitney *U*-test, *p* > 0.05). For the long-delay positive peak, (b) we analyzed *n* = 846 passive VCs (amplitude of 1 [0.034 0.034]) and *n* = 354 active VCs (amplitudes of 0.46 [0.067 0.060]). This difference turned out to be highly significant (Mann-Whitney *U*-test, *p* < 0.01). Figure 2C shows the mean evoked LFPs for active vs. passive cases separately for the three animals. The short-latency negative deflection appears as a kink in the average evoked LFP trace (arrow heads). Also, note that average-evoked LFP traces observed in active vs. passive cases overlap for a few milliseconds following stimulation (end of overlap is pointed to by arrows).

**Figure 3:**
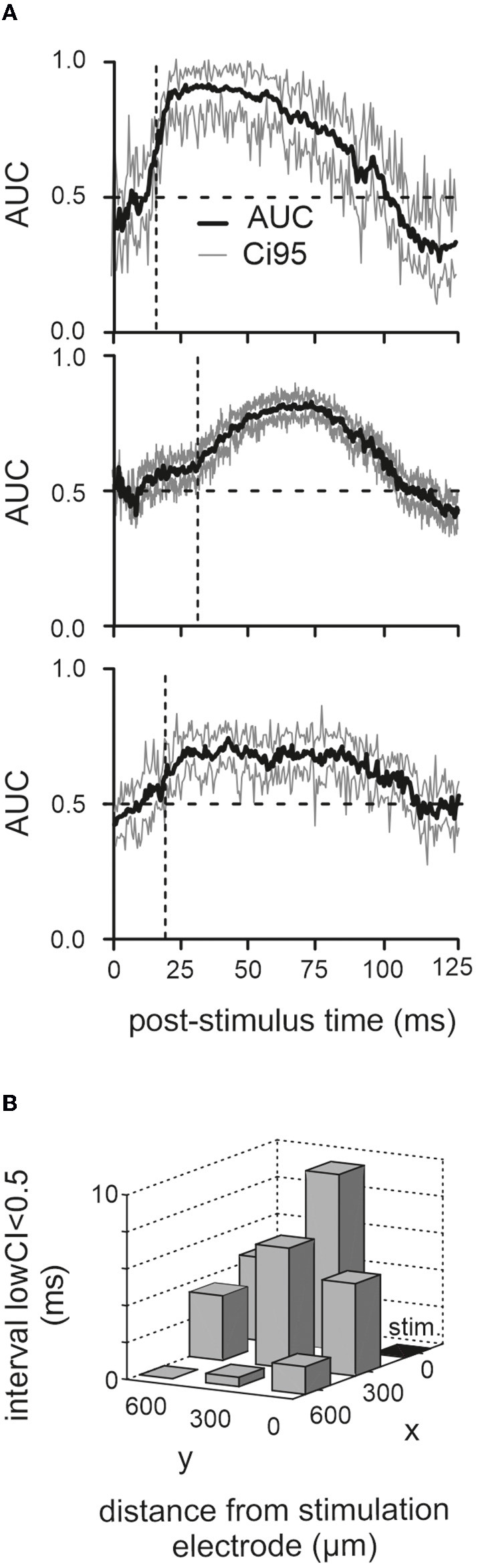
Poststimulus period of nondiscriminability. **(A)** Corresponding mean and CI95 (1,000 bootstraps) of AUC traces computed from the LFP responses in the three animals (the one with the cut infraorbital nerve is shown on top). **(B)** Dwell time of lower confidence border below 0.5 (the time from stimulation time to the first occurrence of lower CI > 0.5 is plotted here). Results from recordings from eight electrodes across all three animals are shown. The stimulation electrode (“stim”) is marked black.

The analysis presented up to here seems to indicate that the different parts of the response, short-latency excitatory vs. long-lasting inhibition, display different susceptibilities for the movement-dependent modulation. This is distinct from experiments performed with more naturalistic whisker touch with an object, where the short-latency neuronal activation volley conveyed *via* the ascending tactile pathway is already susceptible to modulation, and the modulatory signal is related to whisker movement setting some tens of milliseconds before the touch (Hentschke et al., 2006; Chakrabarti and Schwarz, 2018). As electrical stimulation may drive network activity into saturation, modulation may not be possible shortly after electrical activation. We investigated this possibility by calculating the time course of the discriminability of evoked LFP responses in active vs. passive cases across time. Discriminability was quantified as the effect size AUC (same data as in Figure 2C). The result for the three animals is depicted in Figure 3A (1,000 resamples for each time point after stimulation; time resolution 1 ms). In all three animals, the AUC trace was around 0.5 at the time of stimulation. The time point when active and passive traces became discriminable was defined as the time the lower bound of the CI settles above 0.5 at 16, 32, and 18 ms (i.e., vertical broken lines) to give rise to the positive LFP wave (i.e., in the passive case). Although these periods of insensitivity to modulation were different in the three animals, they always included the short-latency negative LFP deflection, consistent with our analysis shown in Figure 2B. We reasoned that if the phenomenon of discriminability is due to network saturation, then the effect should be dependent on the distance of the recording site to the stimulation site. In this case, we would expect that the period of nondiscriminability would be shorter farther away from the stimulation site. This was the case, as shown by the decrement of the time point, the CI first rose above 0.5 with distance from the stimulation electrode (Figure 3B).

## Discussion

In this study, we presented evidence that the local effects of cortical microstimulation are readily modulated by the behavioral state of the animal. There was, however, a clear difference to the modulation as seen with sensory stimulation in previous studies. Immediately after the stimulation including the period of the excitatory burst of spikes (or the short-latency negative peak in the LFP respectively), no modulation by the behavioral state could be observed. Only after a couple of ms, the difference in LFP evoked during active vs. passive cases was manifested. This period of nondiscriminability is best explained by saturation of neuronal circuits as it was decreased with the distance of the recording electrode from the stimulation site and, thus, the amplitude of the evoked LFP response.

### Modulation Dynamics of Stimulation-Evoked Activity

Modulation of the electrically evoked signal, different from sensory signals originating from whisker deflections, only occurs sometime after the first volley of evoked spikes: A period of tens of milliseconds after microstimulation was devoid of signs of gating. Only thereafter, response features were affected. This is consistent with the previous finding that neuronal oscillations, arriving at much longer latencies after stimulation, are suppressed as well (Venkatraman and Carmena, 2009). A possible reason for these dynamics is a saturation of axons and synapses close to the stimulation site. However, before discussing this scenario, we first wish to mention an alternative possibility, namely, that modulation dynamics arise from latencies in neuronal loops. If modulation does not originate from the barrel cortex but happens at a remote site, to which barrel cortex is reciprocally connected, the evoked signals may first be conveyed to the site of modulation and then travel back in a modulated form to the barrel cortex. The latency of such a neuronal loop may then be the basis of the modulation dynamics as observed here. We think this mechanism is rather unlikely mainly because it has been shown that motor signals (or movement-related signals) are present in basically all ascending tactile neuronal structures (Ferezou et al., 2006; Hentschke et al., 2006; Lee et al., 2008; Chakrabarti and Schwarz, 2018). Therefore, the principal mechanistic elements to do sensory gating (i.e., convergent presence of motor or movement-related signals and sensory signals) are present throughout the ascending pathway. On the level of the trigeminal nuclei, the earliest central tactile structure, such movement-related signals stem mainly from the parietal cortex. Once these connections are inactivated, sensory gating is impaired (Chakrabarti and Schwarz, 2018). In ventero-posterior-medial thalamus (VPM), gating is absent if sensory signals are electrically evoked from the lemniscal tract (the direct inputs to VPM) (Lee et al., 2008). Together, these experimental observations strongly suggest that the origin of gating signals is located on the cortical level. One obvious source of movement-related signals is the whisker-related motor cortex (Hill et al., 2011; Gerdjikov et al., 2013), which, in terms of axonal projections, is tightly interconnected with the barrel cortex (Mao et al., 2011). In sensory gating (i.e., active touch), movement-related signals are known to arrive long before any active touch event. That is why sensory gating in the whisker and arm-related tactile systems in rodents and monkeys have been observed to be immediate (relative to the sensory input). That is, even the earliest component of the ascending tactile signal was modulated (Chapman et al., 1988; Hentschke et al., 2006). One of our rats was subjected to the cutting of infraorbital nerves to confirm that we dealt here with the same central gating signals as reported earlier.

In the earlier text, we have discussed the “gating signal” typically arriving together with the movement (i.e., likely consisting of either a motor command or a motor-related signal). Using ICMS in this study, we observed that the gating signal is inactivated shortly after the stimulation. At this point, it is important to differentiate and clarify possible roles of motor structures (e.g., primary motor cortex) beyond gating. Independent of the generation of gating signals, the stimulation-evoked long-latency inhibition (i.e., positive LFP wave) could also be caused by the motor cortex. The motor cortex is known to be intricately interconnected with the barrel cortex, and it has been shown that motor cortex-evoked inhibition of barrel cortex circuitry is possible *via* direct and indirect connections (Kinnischtzke et al., 2014; Audette et al., 2018; Sermet et al., 2019). However, an important observation is that slow inhibition is also observed after electrical stimulation in *in vitro* preparations. The exact origin of the positive LFP wave (or corresponding spike suppression) is therefore not clear. Motor structures are likely to contribute, but local network components alone already give rise to the basic phenomenon (refer to the detailed discussion of this issue in Butovas and Schwarz, 2003, and Butovas et al., 2006).

This finding of modulation kicking in only tens of milliseconds after barrel cortex stimulation thus strongly contrasts the properties of gating found with peripheral sensory signals. Importantly, the absence and presence of modulation is a function of poststimulus time rather than of polarity of the LFP. The time series of AUC values clearly show that the nondiscriminability of LFP signals extends beyond the negative peak well into the positive phase. To understand the phenomenon, it seems important to consider that the fast-excitatory response after peripheral sensory stimulation is generated by the ascending activity in the tactile pathway, while the one of similar appearance observed with microstimulation is due to the little understood mechanism by which neurons are activated using extracellular current in the cortical tissue (Butovas and Schwarz, 2003). It is very likely that neurons in the vicinity of the electrode are activated by direct (i.e., antidromic) as well as indirect (i.e., orthodromic) trans-synaptic mechanisms. Two-photon calcium imaging of spike-related calcium transients revealed that directly activated neurons are distributed sparsely, and the group of activated neurons changes dramatically when the tip of the stimulation electrode moves a small distance of some tens of micrometers (Histed et al., 2009). This result pointed to a few axons around the electrode tip that are initially activated supporting a long-held notion from chronaxie measurements that it is axonal structures and not somas that are the immediate targets of microstimulation (Ranck, 1975; Tehovnik, 1996; Nowak and Bullier, 1998a,b). As axonal membranes are unlikely to be influenced by top-down signals, the predominant role of axons in direct activation by microstimulation may partly explain our observation that immediately after the stimulation, the response is unaffected by top-down responses.

However, direct activation only accounts for a very short period, presumably not exceeding a millisecond after the stimulation pulse. The rest of the neuronal (and behavioral) response is likely based on trans-synaptic conveyance of the activity. Postsynaptic potentials in the vicinity of cortical microstimulation have been measured to arrive at latencies up to 2 ms in the motor cortex of halothane-anesthetized cats (Asanuma and Rosen, 1973), and similar latencies were measured from evoked spikes in the somatosensory cortex of ketamine anesthetized rats (Butovas and Schwarz, 2003). In the latter study, the peak of the spike density was very narrow (<2 ms), and around 90% of neurons responded to the stimulation in an area of 900 μm around the stimulation site, indicating the presence of a massive and highly synchronous volley of trans-synaptic excitation.

The short-latency negative peak observed in LFP measurements in this study corresponds in latency and duration very well to the trans-synaptic excitatory activation of local neurons. In view of the high temporal precision of the evoked spikes it, most likely, corresponds to a population spike, as supported by occasional spike recordings (cf. Figure 1D). The massive nature of the trans-synaptic response renders it likely that the absence of modulation a few milliseconds after the microstimulation reflects a transient saturation of the neurons' responses. Strong evidence in favor of this idea has been obtained by repetitive intracortical microstimulation. The excitatory spike response evoked by repetitive stimulus pulses is not diminished compared with that of single-pulse stimulation, despite the presence of massive inhibitory activity in the repetitive case, arguing strongly for a saturation phenomenon at the time of the short-latency excitatory peak (Butovas and Schwarz, 2003, their Figure 11). In this study, using LFP signals, we found that the period of nonmodulation persists beyond the excitatory response and that it is dependent on distance and, thus response intensity, from the stimulation site. It is therefore likely that the presumed saturation process involves the initial parts of inhibitory action following the first wave of excitation as well (Butovas and Schwarz, 2003; Butovas et al., 2006; Logothetis et al., 2010).

### Implications for the Development of Cortical Neuroprostheses

Behavior-dependent modulation of cortical responses as demonstrated here certainly has to be considered when applying cortical microstimulation for cortical neuroprostheses. If context matters, its neuronal reflections have to be assessed before precise stimulation can be achieved. However, monitoring all possibly relevant signals in the brain is unattainable. In a recent study, we have provided proof of principle that using the local LFP as an estimate of the relevant context may be feasible (Brugger et al., 2011). This study has shown that local LFP in a 20 ms prestimulation interval contains enough information to adapt the stimulus in real-time, such that response variability across trials could be reduced as compared with “blind” stimulation with constant intensity. These previous results together with the present ones point to context-dependent microstimulation as a promising novel way to imprint sensory signals into cortical networks. An important next step is to demonstrate the perceptual effects of context-dependent modulations and demonstrate that context-sensitive dynamic brain stimulation improves it.

## Data Availability Statement

The datasets presented in this article are not readily available because they consist in multichannel systems files, and custom Matlab files. Requests to access the datasets should be directed to cornelius.schwarz@uni-tuebingen.de.

## Ethics Statement

The animal study was reviewed and approved by Regierungspräsidium Tübingen.

## Author Contributions

SB and CS conceived the project and designed experiments, did the analysis, and wrote the article. SB performed the experiments. Both authors contributed to the article and approved the submitted version.

## Funding

This work was supported by DFG SCH577/9-1 and SCH577/16-1.

## Conflict of Interest

The authors declare that the research was conducted in the absence of any commercial or financial relationships that could be construed as a potential conflict of interest.

## Publisher's Note

All claims expressed in this article are solely those of the authors and do not necessarily represent those of their affiliated organizations, or those of the publisher, the editors and the reviewers. Any product that may be evaluated in this article, or claim that may be made by its manufacturer, is not guaranteed or endorsed by the publisher.
